# Legacy Duty—The Rights of the Voluntary Hospitals

**Published:** 1913-03-01

**Authors:** R. Martin Bates

**Affiliations:** Perth Royal Infirmary, Secretary's Office, 44 Tay Street, Perth


*To the Editor of* The Hospital.

### Legacy Duty—The Rights of the Voluntary Hospitals.

*To the Editor of* The Hospital.

Sir,—I read with interest the article in The Hospital of February 22 regarding the relief to hospitals from payment of legacy duty on bequests to them.

I observe that in the last paragraph of the reply to the communication addressed to the Chancellor of the Exchequer regarding this subject, it is pointed out that "It is always open to a testator to bequeath a legacy to a hospital 'free of duty,' and in this way to shift the incidence of duty from the hospital to the residuary legatee."

In many cases, however, a testator bequeaths the residue of an estate to a hospital, in which case the payment of duty at a rate of 10 per cent. must be borne by the institution.

Most excellent reasons are set forth in your article why voluntary hospitals should be relieved of this burden. It should no longer be asked as a concession but demanded as a right. The Chancellor of the Exchequer is under a deep obligation to the voluntary hospitals of this country, without whose aid all efforts to carry out the provisions of the National Insurance Act so far as these relate to medical benefit would be in vain, and the Government should therefore welcome the opportunity of assisting the managers of voluntary hospitals in the laudable desire to relieve their funds of this unnecessary and oppressive burden. In fact it seems to me that the matter only requires to be brought under the notice of the members of Parliament to ensure for it immediate attention.

Perhaps the British Hospitals Association would again circularise the managers of each hospital in the country asking them to lay the facts before their representatives in Parliament.—Yours faithfully, R. Martin Bates.

Perth Royal Infirmary,
Secretary's Office, 44 Tay Street, Perth,
February 24, 1913.

